# Single-center clinical experience of cyanoacrylate embolization method for incompetent perforating veins in treating CEAP-6 patients

**DOI:** 10.1016/j.jvsv.2024.101939

**Published:** 2024-07-01

**Authors:** Ufuk Türkmen

**Affiliations:** Department of Cardiovascular Surgery, Hitit University Faculty of Medicine, Corum, Türkiye

**Keywords:** Cyanoacrylate, Incompetent perforating vein, Embolization, CEAP-6, Venous leg ulcer

## Abstract

**Objective:**

The most severe form of chronic venous insufficiency includes venous leg ulcers in the CEAP-6 stage. The aim of this study is to evaluate the relationship between incompetent perforator veins occluding with cyanoacrylate and closure of perforator veins and healing of venous leg ulcers in patients at the CEAP-6 stage.

**Methods:**

A total of 187 patients who underwent cyanoacrylate application to incompetent perforator veins due to venous leg ulcers from 2018 to 2021 were retrospectively reviewed. Twelve months after the procedure, patients were evaluated for perforator vein closure, ulcer diameter, and Venous Clinical Severity Scale. Receiver operating characteristic analysis was used to estimate the probability of postoperative nonocclusion of the perforating vein based on the preoperative ulcers’ diameters and the perforating veins’ mean diameters. Univariate and multivariate binary logistic regression analyses were conducted to identify the risk factors associated with incomplete closure of the perforating vein.

**Results:**

At the 12 months, 87.1% of patients experienced incompetent perforator veins closure, leading to complete healing of venous leg ulcers. Preoperative ulcer diameter significantly decreased from 7.20 ± 3.48 cm^2^ to 0.28 ± 0.77 cm^2^ after the procedure (*P* < .001). On average, 3.5 ± 1.01 perforating veins were treated, with a diameter of 4.09 ± 0.41 mm. No postoperative paresthesia or deep vein thrombosis occurred. Preoperative Venous Clinical Severity Scale scores decreased significantly from 17.85 ± 3.06 to 8.03 ± 3.53 postoperatively (*P* < .001). Patients with nonoccluded perforating veins had larger preoperative ulcer diameters (13.77 ± 1.78 cm^2^) than those with occluded perforating veins (6.24 ± 2.47 cm^2^; *P* < .001). The mean perforating vein diameter was also larger in nonoccluded perforating veins patients (4.45 ± 0.41 mm) than in occluded perforating veins patients (4.04 ± 0.38 mm; *P* < .001). The sensitivity, specificity, and accuracy of the preoperative ulcer diameter cutoff point of 11.25 cm^2^ for the possibility of postoperative nonocclusion of perforating veins were 100% each. In contrast, those for the preoperative mean perforating vein diameter cutoff point of 4.15 mm were determined as 66.7%, 79.1%, and 77.5%, respectively. The presence of diabetes mellitus increased the likelihood of incompetent perforator veins, remaining open by 3.4 times (95% confidence interval: 1.11-10.44; *P* = .032), whereas a 1 mm larger mean perforating vein diameter increased this likelihood by 9.36 times (95% confidence interval: 3.47-25.29; *P* < .001).

**Conclusions:**

This study demonstrates that occlusion of incompetent perforator veins with cyanoacrylate is effective, safe, and associated with low complication rates in CEAP-6 patients. The findings support that cyanoacrylate occlusion of perforator veins may be a valuable option in the treatment of venous leg ulcers.

**Article Highlights**

• **Type of Research:** Single-center, retrospective cohort study
• **Key Findings:** At 12 months, the rate of incompetent perforating vein (IPV) closure and consequent venous leg ulcer healing was found to be 87.1% in 187 CEAP-6 patients who underwent IPV closure with cyanoacrylate. The receiver operating characteristic analysis conducted to predict the likelihood of perforator veins remaining open during the postoperative period yielded a cutoff value of 11.25 cm^2^ for preoperative ulcer size and 4.15 mm for preoperative perforator vein diameter. The likelihood of IPV remaining open was found to be 3.4 times higher in individuals with diabetes mellitus disease and 9.36 times higher with a 1 mm increase in mean perforator vein diameter. The Venous Clinical Severity Score decreased from 17.85 to 8.03, and no patients experienced deep vein thrombosis.
• **Take Home Message:** The closure of IPV with cyanoacrylate in CEAP-6 patients has provided a minimally invasive treatment option for wound healing.

According to the CEAP (Clinical, Etiological, Anatomical, and Pathophysiological) classification, the most severe form of chronic venous insufficiency (CVI) is the CEAP-6 stage, which includes active venous leg ulcers (VLUs).^1^ Affecting approximately 1% of the adult population, VLU reduces patients’ quality of life, and its treatment imposes a significant cost on countries’ health systems.^2^

After establishing the relationship between incompetent perforator veins (IPVs) and VLU, the treatment approach initially involved surgical ligation of IPVs, which later evolved into subfascial endoscopic perforator surgery.^3^, ^4^, ^5^ With the widespread adoption of ultrasound examination in clinical settings, subfascial endoscopic perforator surgery was subsequently replaced by minimally invasive techniques such as radiofrequency endovenous ablation), endovenous laser ablation, sclerotherapy, and cyanoacrylate techniques.^6^, ^7^, ^8^, ^9^

In patients with isolated pathological perforating veins under or associated with a healed (CEAP-5) or active ulcer (CEAP-6) area, regardless of deep vein status, the ablation of pathological perforating vessels is recommended in addition to standard compression therapy to aid venous ulcer healing and prevent recurrence. Although thermal ablation techniques successfully treat IPVs, they require anesthesia and may unintentionally cause nerve damage.^10^

Cyanoacrylate is a liquid embolizing agent used for many years to treat venous insufficiency. It does not require thermal energy and thus avoids the risk of thermal nerve injury. It solidifies on contact with blood, causing a rapid inflammatory reaction.^9^^,^^11^^,^^12^

This study evaluated the relationship between IPV and VLU healing after endovenous occlusion of IPVs with cyanoacrylate in CEAP-6 patients who received optimal treatment with compression and wound care for at least 3 months.

## Methods

This study included 187 patients who presented to the Hitit University Faculty of Medicine Cardiovascular Surgery Clinic between January 2018 and December 2021 and underwent cyanoacrylate occlusion for IPVs at the CEAP-6 stage. Institutional approval for the study was obtained from the Hitit University Faculty of Medicine Ethics Committee (decision no: 2023-35), following the principles of the Declaration of Helsinki. Individual informed consent for IPV treatment was obtained from all patients.

Patients’ data were retrospectively analyzed from electronic medical record databases and patient files. Demographic information, preoperative and postoperative 12th-month IPV, and wound diameters, as well as Venous Clinical Severity Score (VCSS)^13^ data, were recorded for each patient. When determining ulcer diameters, the longest and widest dimensions of the wound were taken into account.

All patients presenting with VLU underwent routine venous Doppler ultrasound (DUSG) examinations of the deep and superficial venous systems, as well as perforator veins, performed by a radiologist. Perforator veins with a diameter of at least 3.5 mm and a reflux duration of at least 500 ms were considered incompetent.^14^ All patients have only undergone intervention for their IPVs, and no procedure has been performed on all other lower extremities veins. The term “deep vein thrombosis (DVT) history” refers to a resolved thrombus detected via DUSG in the iliac, femoral, or popliteal veins, whereas “deep vein insufficiency (DVI)” denotes insufficiency in these veins.

The inclusion criteria were as follows: patients older than 18 years, having at least one VLU in the lower extremity, detection of at least one perforating vein insufficiency by DUSG, no improvement in VLU despite wound care and compression therapy for at least 3 months, and patent arterial vessels on DUSG.

The exclusion criteria were as follows: non-VLU (diabetic and arterial), VLU lasting less than 3 months, the absence of regular weekly wound care and compression therapy, lack of ulcer measurement, arterial disease detected on DUSG, unresolved DVT obstruction at any deep venous level, history of cancer, systemic inflammatory diseases, and pregnancy.

### Procedure

One surgeon performed all procedures in the operating room under sterile conditions using B-Mode and DUSG on the LOGIQ (Ultrasound; GE Healthcare Technologies). Patients received routine antibiotic prophylaxis before IPV occlusion. In all patients, VariClose’s cyanoacrylate glue (Biolas Inc) with a polymerization time of 3 to 4 seconds was used.^15^ The lower extremities of the patients to be intervened were sterilized with a 10% povidone-iodine solution. The operating table was positioned in the reverse Trendelenburg position to facilitate percutaneous access to the IPVs. With a sterile cover placed on the DUSG probe and sterile gel applied during the procedure, all targeted perforating veins were identified. If access to the IPV beneath the active wound tissue was feasible from the sides of the wound, the procedure was performed; direct intervention over the active wound was avoided. The DUSG probe was longitudinally employed to enhance visualization of the needle in the perforating vein targeted for intervention. Access to perforating veins was achieved through intact skin tissue. Approximately 0.5 cc of 1% lidocaine was injected at the procedural site. Two-cubic centimeter syringes prefilled with 0.5 mL of cyanoacrylate for the planned number of perforating veins were used. To prevent polymerization on contact with blood, cyanoacrylate was ensured not to leak from the needle tip. A 21-gauge needle was inserted longitudinally into the midpoint of the IPVs under DUSG guidance. Once the correct position was reached, cyanoacrylate was swiftly injected to prevent polymerization at the needle tip, and injection was confirmed by DUSG. Subsequently, compression was applied for approximately 10 seconds. This process was repeated for each IPV. After confirmation of no flow in the IPVs on DUSG and observation of the cyanoacrylate hyperechoic image in the occluded IPV, the extremities from the feet to the knees were wrapped with an elastic bandage. After the procedure, patients were monitored for a few hours and then discharged with antibiotics tailored to each patient. No patient received low-molecular-weight heparin for venous thromboembolism prophylaxis. After removal of the elastic bandage after 24 hours, treatment continued with closed dressing and daily compression stockings with 20 to 30 mm Hg pressure. Throughout the 12-month follow-up period, no patient required reintervention on IPVs. The VCSS at the 12th month was used to objectively evaluate clinical improvement. The final end point was the occlusion status of the IPV and the final condition of the VLU compared with the preoperative state.

### Statistical methods

Statistical analyses were conducted using SPSS software (version 22; SPSS Inc). Descriptive statistics for categorical variables were presented as frequency (n) and percentage (%). Relationships between categorical variables were assessed using either the χ^2^ test or the Fisher exact test. Descriptive statistics for numerical data were reported as mean ± standard deviation for normally distributed data and median (minimum-maximum) with mean ± standard deviation for non-normally distributed data. Normal distribution assumption was tested using the Kolmogorov-Smirnov test, Shapiro-Wilk test, and graphical methods. The assumption of homogeneity of variances was tested using Levene’s test. Comparison of numerical data between two independent groups was performed using the Student *t* test for parametric test assumptions and the Mann-Whitney *U* test for nonparametric test assumptions. Comparison of numerical data before and after was conducted using the paired *t* test for parametric test assumptions and the Wilcoxon signed-rank test for nonparametric test assumptions.

Receiver operating characteristic (ROC) analysis was used to determine prognostic indicators for predicting perforator vein patency. The ROC area under the curve (AUC) and its 95% confidence intervals (CIs) were calculated. AUC values obtained from ROC analysis were interpreted as follows: 0.5 to 0.6: unsuccessful, 0.6 to 0.7: poor, 0.7 to 0.8: moderate, 0.8 to 0.9: good, and 0.9 to 1: excellent. Optimal cutoff points were determined using the Youden index. Sensitivity, specificity, accuracy, positive predictive value, and negative predictive value were calculated. A significance level of *P* < .05 was considered for all statistical comparisons.

Univariate and multivariate binary logistic regression analyses were performed to assess predictors of perforator vein patency. Parameters significant at the 0.15 level in the univariate model were included in the multivariate model. The final model included parameters significant at the 0.05 level. Odds ratios (ORs) with 95% CIs were calculated for each significant parameter in univariate and multivariate logistic regression models.

## Results

In the study, data from 187 patients, comprising 61 (32.6%) women and 126 (67.4%) men, were subjected to statistical analysis. At the end of 12 months, IPV closure was achieved in 163 patients (87.1%), resulting in complete healing of associated VLUs. The mean preoperative ulcer diameter of the patients (7.20 ± 3.48 cm^2^) decreased significantly after the operation (0.28 ± 0.77 cm^2^; *P* < .001). The average number of treated perforating veins was 3.5 ± 1.01. The average diameter of perforating veins was calculated as 4.09 ± 0.41 (mm). Descriptive statistics of the sociodemographic and some clinical characteristics of the patients are presented in Table I.

**Table I:** Descriptive statistics of sociodemographic and clinical characteristics of the patients and statistical findings for the comparison of the preoperative and postoperative Venous Clinical Severity Score (*VCSS*)

Characteristic	Descriptive statistics (n = 187)
Gender	
Female	61 (32.6)
Male	126 (67.4)
Smoking	
No	108 (57.8)
Yes	79 (42.2)
DM	
No	153 (81.8)
Yes	34 (18.2)
Previous saphenous vein surgery	
No	131 (70.1)
Yes	56 (29.9)
DVT history	
No	62 (33.2)
Yes	125 (66.8)
DVI	
No	108 (57.8)
Yes	79 (42.2)
Age	53.42 ± 13.69
BMI	29.52 ± 2
Preoperative ulcer diameter, cm^2^	6 (1-18)
	7.20 ± 3.48
Postoperative ulcer diameter, cm^2^	0 (0-4)
	0.28 ± 0.77
No. of perforating veins operated	3 (2-6)
	3.5 ± 1.01
Mean perforating vein diameter, mm	4 (3.5-5)
	4.09 ± 0.41

Preoperative VCSS	Postoperative VCSS	*P* values
17.85 ± 3.06	8.03 ± 3.53	<.001^a^

*BMI*, Body mass index; *DM*, diabetes mellitus; *DVI*, deep venous insufficiency; *DVT*, deep vein thrombosis.

Descriptive statistics were reported using mean ± standard deviation or median (minimum-maximum) depending on the data normal distribution for continuous variables and number and percentage (%) for categorical variables.

a Paired *t* test with mean ± standard deviation.

No postoperative paresthesia or newly developed DVT was observed in any patient. Although phlebitis at the site of intervention was observed in only seven patients, it regressed with antibiotic treatment.

**Fig 1:**
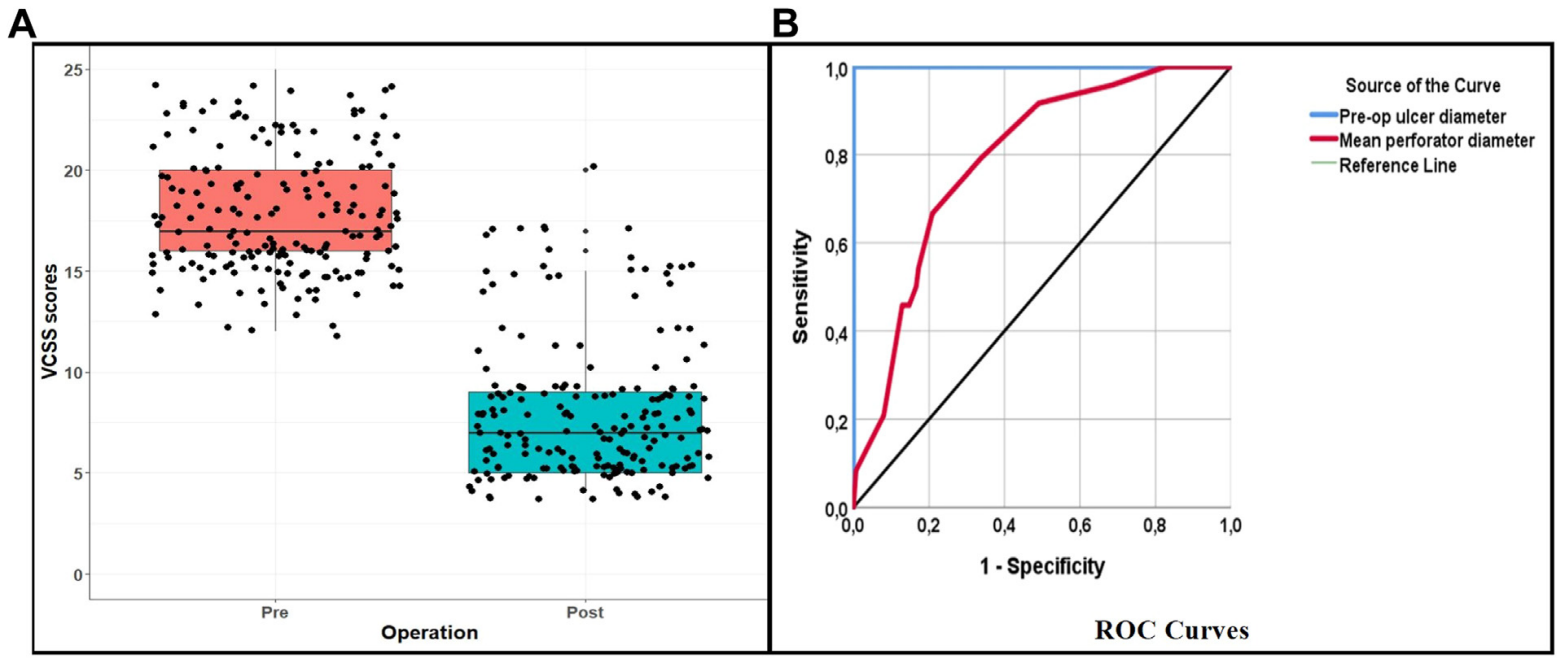
**A,** Box plot with jitters showing preoperative and postoperative Venous Clinical Severity Score (*VCSS*) distributions. **B,** Receiver operating characteristic (*ROC*) curves showing the success of preoperative ulcer diameter and mean perforator diameter parameters in estimating perforator vein patency.

Statistical analysis revealed a significant decrease in patients’ preoperative VCSS (17.85 ± 3.06) compared with postoperative scores (8.03 ± 3.53; *P* < .001, Fig 1, *A*, Table I).

After 12 months of follow-up, statistical findings comparing sociodemographic and some clinical characteristics between patients with nonoccluded perforating veins (N-OPVs) (n = 24) and patients with fully occluded perforating veins (OPVs) (n = 163) are presented in Table II. There was no significant difference in the mean age, body mass index (BMI), gender, smoking, diabetes mellitus (DM), previous saphenous vein surgery, DVT history, and DVI between the groups. The mean preoperative ulcer diameter of patients with N-OPV (13.77 ± 1.78 cm^2^) was significantly higher than the mean preoperative ulcer diameter of patients with OPV (6.24 ± 2.47 cm^2^; *P* < .001). The mean perforating vein diameter of patients with N-OPV (4.45 ± 0.41 mm) was significantly higher than the mean perforating vein diameter of patients with OPV (4.04 ± 0.38 mm; *P* < .001).

**Table II:** Statistical findings for the comparison of some risk factors between patients with occlude and nonoccluded perforator veins

Characteristic	Perforator vein		*P* values
	Occluded (n = 163)	Nonoccluded (n = 24)	
Age	53.41 ± 13.93	53.46 ± 12.18	.987^a^
BMI	29.39 ± 1.86	30.37 ± 2.66	.096^a^
Preoperative ulcer diameter, cm^2^	6.24 ± 2.47	13.77 ± 1.78	<.001^a^
Mean perforator diameter, mm	4.04 ± 0.38 3.9 (3.5-5)	4.45 ± 0.41 4.35 (3.8-5)	<.001^b^
Gender			
Male	108 (66.3)	18 (75)	.394^c^
Female	55 (33.7)	6 (25)	
Smoking			
No	93 (57.1)	15 (62.5)	.614^c^
Yes	70 (42.9)	9 (37.5)	
DM			
No	136 (83.4)	17 (70.8)	.157^d^
Yes	27 (16.6)	7 (29.2)	
Previous saphenous vein surgery			
No	113 (69.3)	18 (75)	.571^c^
Yes	50 (30.7)	6 (25)	
DVT history			
No	54 (33.1)	8 (33.3)	.984^c^
Yes	109 (66.9)	16 (66.7)	
DVI			
No	94 (57.7)	14 (58.3)	.951^c^
Yes	69 (42.3)	10 (41.7)	

*BMI*, Body mass index; *DM*, diabetes mellitus; *DVI*, deep venous insufficiency; *DVT*, deep vein thrombosis.

a Student’s *t* test with mean ± standard deviation (SD).

b Mann-Whitney *U* test mean ± SD with median (minimum-maximum).

c χ^2^ test with number (%).

d Fisher’s exact test with number (%).

**Fig 2:**
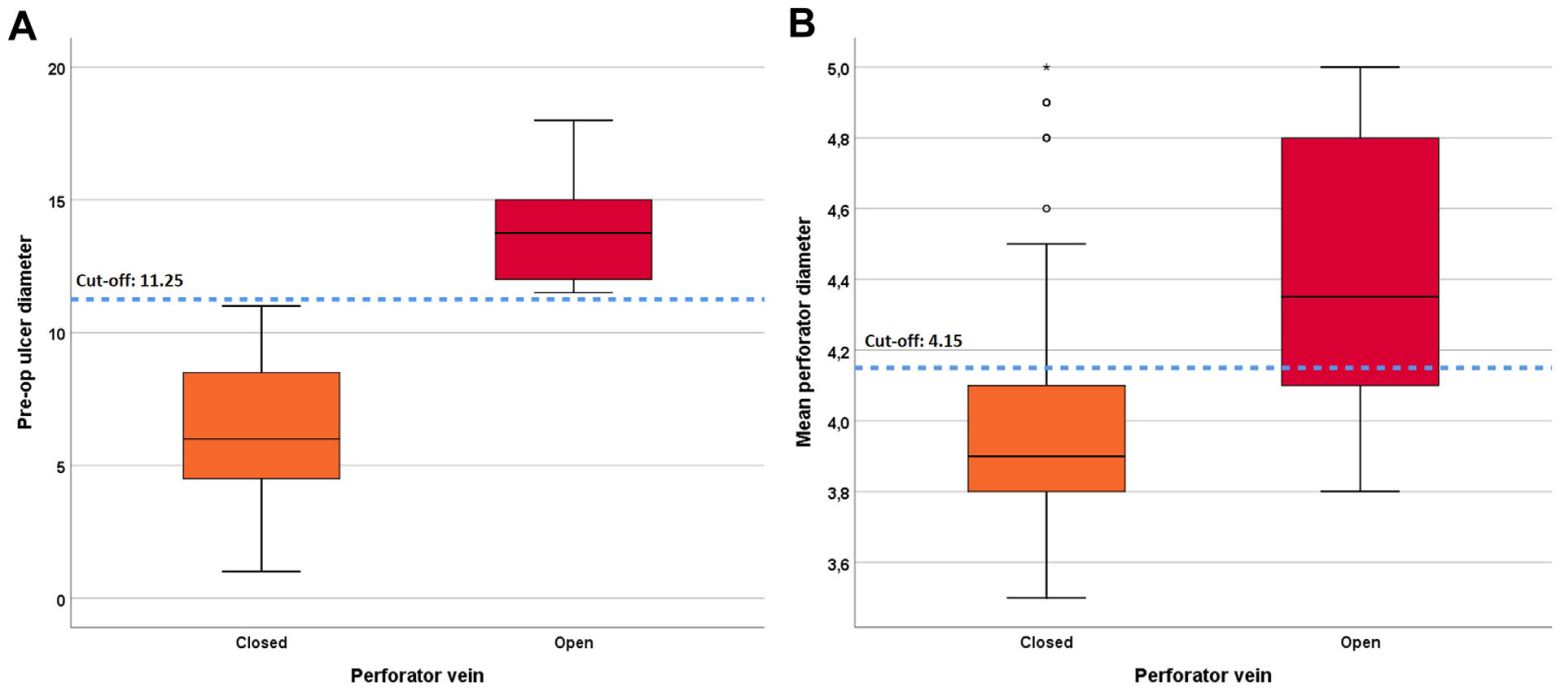
Box plots showing the success of the cutoff points determined by receiver operating characteristic (ROC) analysis for the preoperative ulcer diameter and mean perforator diameter parameters in estimating perforator vein patency.

The sensitivity, specificity, accuracy, and positive and negative predictive values were calculated to determine the success rates of the cutoff values determined as a result of the ROC analysis performed to predict whether the perforating vein may remain open from preoperative ulcer diameters and average perforating vein diameters, and they are presented in Table III. According to the ROC analysis results, preoperative ulcer diameters and average perforating vein diameters were found to be highly and moderately significant, respectively, in predicting whether the perforating vein would remain open (AUC = 1 [1-1], AUC = 0.789 [0.704-0.874], respectively; Table III). The classification success for the 11.25 cutoff point determined for the preoperative ulcer diameter was 100% sensitivity (95% CI: 83-100), 100% specificity (95% CI: 97-100), and 100% accuracy. The classification success for the 4.15 cutoff point determined for the average perforating vein diameter was 66.7% sensitivity (95% CI: 44.7-83.6), 79.1% specificity (95% CI: 71.9-84.9), and 77.5% accuracy. The ROC curve obtained for preoperative ulcer diameters and average perforating vein diameters in predicting whether the perforating vein will remain open is presented in Fig 1, *B*, and the box plots showing the success of the cutoff points are presented in Fig 2. In addition, the findings showing the classification success according to the cutoff points obtained by ROC analysis are presented in Table III.

**Table III:** Sensitivity, specificity, positive predictive value (*PPV*), negative predictive value (*NPV*), and likelihood ratio (+) values of preoperative ulcer diameter and mean perforator diameter in the prediction of perforator vein patency

	Preoperative ulcer diameter	Mean perforator diameter
AUC (95% CI)	1 (1-1)	0.789 (0.704-0.874)
Cutoff	≥11.25	≥4.15
Accuracy, %	100	77.5
Sensitivity (95% CI), %	100 (83-100)	66.7 (44.7-83.6)
Specificity (95% CI), %	100 (97-100)	79.1 (71.9-84.9)
PPV (95% CI), %	100 (83-100)	32 (19.9-46.8)
NPV (95% CI), %	100 (97-100)	94.2 (88.4-97.3)

	Cutoff	Perforator vein		Total
		Occluded	Nonoccluded	
Preoperative ulcer diameter	<11.25	163	0	163
	≥11.25	0	24	24
Mean perforator diameter	<4.15	129	8	137
	≥4.15	34	16	50
Total		163	24	187

*AUC*, Area under curve; *CI*, confidence interval; *ROC*, receiver operating characteristic.

The success of cutoff values determined by ROC analysis in the prediction of perforator vein patency.

The results of the univariate and multivariate binary logistic regression analysis performed to determine the risk factors affecting the incomplete closure of the perforating vein and the ORs and 95% CIs for each statistically significant parameter are given in Table IV. In the univariate model, age, gender, smoking, previous saphenous vein surgery, DVT history, and DVI parameters were statistically insignificant (*P* > .05). In the univariate model, the effect of BMI, DM, and mean perforating vein diameters was significant at the *P* < .15 level (*P* = .029, *P* = .141, and *P* < .001, respectively). The OR (95% CI) was calculated as 1.26 (1.02-1.56) for BMI, 2.07 (0.78-5.48) for DM, and 7.59 (2.99-19.21) for the mean perforating vein diameter in the univariate model (Table IV). The effects of BMI, DM, and average perforating vein diameters were found to be significant at the *P* < .15 level in the univariate model, and the effect of BMI was insignificant at the *P* < .05 level in the multivariate model (*P* = .139). According to the final model results obtained by excluding BMI from the model, the presence of DM increased the probability of the perforating vein remaining open by 3.4 times (95% CI: 1.11-10.44; *P* = .032). A 1 mm larger mean perforating vein diameter increased the probability of the perforating vein remaining open by 9.36 times (95% CI: 3.47-25.29; *P* < .001).

**Table IV:** Results of univariate and multivariate binary logistic regression analysis performed to determine the risk factors that are effective in the prediction of perforator vein patency

	Univariate		Multivariate	
	*P* values	OR (95% CI)	*P* values	OR (95% CI)
Age	.987	–	ni	–
Gender	.396	–	ni	–
Smoking	.615	–	ni	–
Previous saphenous vein surgery	.572	–	ni	–
DVT history	.984	–	ni	–
DVI	.951	–	ni	–
BMI	.029	1.26 (1.02-1.56)	.139	–
DM (yes vs no)	.141	2.07 (0.78-5.48)	.032	3.4 (1.11-10.44)
Mean perforator diameter (>4.15 vs minors)	<.001	7.59 (2.99-19.21)	<.001	9.36 (3.47-25.29)

*BMI*, Body mass index; *CI,* confidence interval; *DM*, diabetes mellitus; *DVI*, deep venous insufficiency; *DVT*, deep vein thrombosis; *ni*, not included; *OR*, odds ratio.

Multivariate model: Nagelkerke *R*^*2*^ = 0.226; classification success: 87.2%.

## Discussion

This study presents an effective method with low rates of recanalization and minimal complications for treating IPV in CEAP-6 patients. CVI affects the venous system of the lower extremities, primarily caused by venous hypertension from valve insufficiency and reflux due to obstruction. CVI encompasses various conditions, leading to pain, edema, skin changes, and ulcers due to increased venous pressure. Compression therapy is crucial in delaying or preventing these issues. Although multilayer dressings are often effective, some ulcers may not heal, requiring further vascular assessment with DUSG and potential surgical intervention. Treatment options aim to address reflux in saphenous or perforator veins, often resulting in ulcer healing. Minimally invasive techniques are preferred due to their lower complication risks.^16^

Local wound care and multilayer compression therapy have continued to be the standard treatment for VLU for years.^10^ Despite the optimal application of this standard treatment, recurrences in VLU are inevitable.^17^ The ESCHAR study followed by the EVRA study demonstrated that surgical treatment of refluxing pathological superficial veins reduces the time required for VLU healing and improves wound healing rates.^18^^,^^19^

In patients with CEAP-6, in addition to compression therapy and wound care for venous reflux resulting in venous hypertension in the lower extremities, intervention is also required for the refluxing vascular structures.^10^ In these patients, it has been proven that thermal and nonthermal minimal invasive endovascular treatment methods are safe and effective for the ablation of refluxing pathological saphenous veins.^19^, ^20^, ^21^

Although ablation methods for the saphenous veins have proven themselves in CEAP-6 patients, approaches to perforator veins in these patients are still limited. Although isolated incompetence of perforator veins is rare, their role in the treatment of VLU and varicose veins remains debatable. Although most studies advocate for the treatment of IPVs as a component of chronic venous disease, some do not agree with this view.^22^, ^23^, ^24^

The majority of endovascular treatments for VLU focus on thermal and nonthermal methods applied to the saphenous veins, or thermal methods applied solely to IPV, or their comparison. Because VLUs are typically located distally in the leg, there is a high likelihood of nerve damage with thermal methods. In addition, applying thermal methods to the most distal IPVs, such as May’s and Kustner’s perforators, may be challenging. The need for tumescent anesthesia in thermal methods and concerns about nerve damage associated with thermal energy have led some researchers to favor nonthermal methods.^19^, ^20^, ^21^^,^^25^, ^26^, ^27^

Cyanoacrylate rapidly polymerizes on contact with ionic solutions like blood, causing vessel occlusion by triggering an inflammatory reaction. Despite being an exothermic reaction, the polymerization process does not damage the endothelium or pose a risk of thermal nerve injury. Cyanoacrylate has been used for many years to treat venous insufficiency and has many successful applications.^11^^,^^12^

The use of cyanoacrylate in treating IPV has a decade-long history. Studies have reported success rates ranging from 76% to 100% in occluding IPV. What sets this study apart is that all patients in the cohort were CEAP-6 patients, unlike previous studies where only a small portion fell into this category. After a 12-month follow-up, the study found an 87.1% rate of IPV closure and subsequent VLU healing. Prasad et al^28^ have linked high occlusion rates in IPV with concurrent foam sclerotherapy, especially when combined with subcutaneous collateral foam sclerotherapy. No additional interventions were undertaken during the 12-month study period besides wound dressing and compression therapy.^9^^,^^28^^,^^29^ Although differences in occlusion rates between studies may imply an association with sclerotherapy, further investigation is warranted through comparative prospective studies.

This study differs from others in that cyanoacrylate was directly applied to the IPV using a 21G needle, eliminating the need for an additional catheter. Guided by DUSG, the needle was inserted longitudinally into the IPV, and cyanoacrylate was injected approximately 1 cm away from the deep veins. The application occurred while withdrawing the needle rapidly, with each patient receiving between 1 mL and 3 mL of cyanoacrylate and 0.5 mL applied to each IPV. Despite using a higher total amount of cyanoacrylate than other studies on IPV and saphenous vein treatment, the short polymerization time of the cyanoacrylate used, the pressure applied to the IPV after each application, and the separation of the IPV from each other prevented the escape of embolic material into the deep veins.^9^^,^^15^^,^^28^^,^^29^ Although concerns about the potential for cyanoacrylate to spread to deep veins, as noted by other authors, were present, no newly developed DVT was encountered during or after the procedure, as confirmed by clinical examination or DUSG.^28^^,^^29^

Cyanoacrylate, being nonabsorbable, may pose infection risks in patients with active VLUs. Hence, preprocedural intravenous antibiotics and a 10-day course of oral antibiotics after the procedure were administered to patients. Although no severe infections were observed, oral antibiotics successfully treated phlebitis in seven cases at the intervention site. This manageable outcome is considered acceptable in high-risk patients with VLU.^30^

The IPV closure procedure initially targeted the longitudinal proximal and distal IPVs of the VLU. Cyanoacrylate was then applied to the IPV surrounding the VLU, if present. At least two IPVs were intervened upon based on VLU size. This study achieved an 87.1% recovery rate, similar to previous studies reporting a 90% recovery rate when at least one IPV was closed.^31^^,^^32^ It was observed that preoperative ulcer and IPV diameters were generally the largest in the 24 patients whose VLU did not completely close at the end of 12 months, although their size decreased.

It has been previously demonstrated that wound size is one of the criteria for healing in VLU, with every 1 cm^2^ increase in wound size leading to a 10% decrease in the probability of wound closure, especially with larger wounds over 10 cm^2^ having lower healing rates.^33^ In this study, the preoperative mean ulcer diameter of patients with N-OPV was 13.77 ± 1.78 cm^2^, whereas that of patients with OPV was 6.24 ± 2.47 cm^2^. The sensitivity, specificity, and accuracy of the preoperative ulcer diameter cutoff point of 11.25 cm^2^ for the possibility of postoperative nonocclusion of perforating veins were each 100% with ROC analysis (Table III, Fig 2). O’Banion et al^21^ classified wounds and defined wounds over 11 cm^2^ as large wounds, and the results of this study were similar.

Identifying preoperative risk factors for N-OPV other than wound size will provide guidance for future studies and clinical use. In the univariate model, BMI showed a significant effect at the *P* < .15 level, whereas in the multivariate model, its effect was not significant at the *P* < .05 level (Table IV). Hager et al^34^ demonstrated a higher association between a BMI >50 kg/m^2^ and N-OPV. In this study, the insignificance of BMI in the multivariate analysis was attributed to the cohort’s average BMI of 29.52 ± 2 (Table I), compared with 30.37 ± 2.66 in the group with N-OPV (Table II). Consistent with these two studies, the increasing BMI suggests a higher rate of nonclosure of IPV.

Aurshina et al^35^ found higher rates of recanalization in perforating veins in the calf region, suggesting that perforator veins in this area are wider, based on clinical experience. Although there is a lack of information in the study regarding this aspect, N-OPVs were analyzed based on preoperative mean perforator vein diameters after cyanoacrylate occlusion. According to the ROC analysis conducted to predict the probability of postoperative nonocclusion of the perforating vein from the preoperative mean perforator vein diameters, a cutoff point of 4.15 mm was determined for the mean perforator vein diameter (Table III, Fig 2).

In addition, two more outcomes were obtained based on the analysis conducted to identify preoperative risk factors in this study. The presence of DM increases the likelihood of perforating veins remaining open by 3.4 times, whereas a 1 mm increase in the preoperative mean perforating vein diameter was associated with a 9.36 times higher probability of perforating veins remaining open (Table IV). The conclusion to be drawn from this is that as the diameter of the IPV increases, along with increasing BMI and the presence of DM, there is a need to increase the volume of cyanoacrylate used or consider repeated procedures.

Another end point in this study was the VCSS values before and at the 12th month after the treatment.^13^ Especially in the VUERT study, values quite similar to the radiofrequency endovenous ablation group were obtained.^36^ This study demonstrated the potential benefit of using VCSS in follow-ups after the treatment of IPV with cyanoacrylate in CEAP-6 patients.

### Limitations

The first limitation of the study is its single-center, retrospective design. The other limitation includes the lack of attention to the wound-healing process. Previous studies have noted that as VLUs persist, healing becomes more challenging.^37^ Despite the known impact of wound chronicity on healing, this study focused on the effectiveness of cyanoacrylate rather than the healing time. Another limitation is the lack of investigation into the impact of resolved DVT and DVI at different venous levels on IPV occlusion across subgroups.

## Conclusions

The direct injection of cyanoacrylate into the IPV under DUSG guidance is a simple procedure that is safe, effective, and associated with low complication rates for occluding pathological perforator vessels in patients with VLU. Despite the low rate of recanalization, repeated injections can be administered to the same area if the IPV does not close. The occlusion of perforators with cyanoacrylate adhesive should be performed by physicians experienced in both cyanoacrylate and DUSG techniques.

## Author Contributions

Conception and design: UT

Analysis and interpretation: UT

Data collection: UT

Writing the article: UT

Critical revision of the article: UT

Final approval of the article: UT

Statistical analysis: UT

Obtained funding: Not applicable.

Overall responsibility: UT

## Disclosures

None.
